# Strategies for Overcoming Resistance in Tumours Harboring *BRAF* Mutations

**DOI:** 10.3390/ijms18030585

**Published:** 2017-03-08

**Authors:** Nourah Mohammad Obaid, Karen Bedard, Weei-Yuarn Huang

**Affiliations:** 1 Department of Pathology, Dalhousie University, Halifax, NS B3H 4R2, Canada; nourah-m-obaid@Dal.Ca (N.M.O.); kbedard@gmail.com (K.B.); 2 Department of Pathology, Nova Scotia Health Authority, Halifax, NS B3H 1V8, Canada

**Keywords:** BRAF, acquired resistance

## Abstract

The development of resistance to previously effective treatments has been a challenge for health care providers and a fear for patients undergoing cancer therapy. This is an unfortunately frequent occurrence for patients undergoing targeted therapy for tumours harboring the activating V600E mutation of the *BRAF* gene. Since the initial identification of the *BRAF* mutation in 2002, a series of small molecular inhibitors that target the BRAFV600E have been developed, but intrinsic and acquired resistance to these drugs has presented an ongoing challenge. More recently, improvements in therapy have been achieved by combining the use of BRAF inhibitors with other drugs, such as inhibitors of the downstream effector mitogen activated protein kinase (MAPK)/extracellular-signal regulated kinase (ERK) kinase (MEK). Despite improved success in response rates and in delaying resistance using combination therapy, ultimately, the acquisition of resistance remains a concern. Recent research articles have shed light on some of the underlying mechanisms of this resistance and have proposed numerous strategies that might be employed to overcome or avoid resistance to targeted therapies. This review will explore some of the resistance mechanisms, compare what is known in melanoma cancer to colorectal cancer, and discuss strategies under development to manage the development of resistance.

## 1. Introduction

Beginning with the discovery of the retroviral oncogene *RAF* (originally named from rapidly accelerated fibrosarcoma) in 1983, the family of *RAF* proto-oncogenes has emerged as an important set of signaling molecules that play a role in the development of cancer [1]. Molecules such as growth factors and cytokines that promote cell proliferation can activate a signaling cascade initiated through receptor tyrosine kinases at the surface and transmit a signal through a series of protein modifications passing from RAS (originally named from rat sarcoma) to RAF to mitogen activated kinase (MAPK) to extracellular signal regulated kinase (ERK) and ultimately to the nucleus, where gene expression changes lead to cell proliferation [2,3]. Shortly after the discovery of the RAF gene family, it was found that activating mutations in the *BRAF* gene, encoding the B-raf serine-threonine protein kinase, play an oncogenic role in cancer development [4]. Currently, it is estimated that eight percent of all cancers have mutations in the *BRAF* gene, and they are present in a wide range of malignant tumours including ~50% of melanomas [5], ~40% of papillary thyroid cancer (PTC) [6], ~30% of serous ovarian cancer [6,7,8,9], ~10% of colorectal cancers (CRC) [10], and ~2%–3% of lung cancers [7,11]. Mutations in *BRAF* have also been found in a pre-malignant colon polyps [12], as well as benign skin lesions [13,14]. This finding of oncogenic mutations in lesions that have not yet advanced to cancer can be attributed to the ability of *BRAF* mutations (like other oncogenic mutations) to induce growth arrest and senescence [15].

To date, more than fifty distinct mutations in the *BRAF* gene have been described [7]. The BRAFV600E mutation accounts for approximately 90% of the *BRAF* mutations found in cancer [1,16,17,18]. This variant (rs113488022) represents an acquired mutation, and is observed only extremely rarely in genomic DNA samples, with a frequency of 0.0016% in the ExAC sequence database. The single nucleotide change from a thymine (T) to adenine (A) at position 1799 of the messenger RNA (NM_004333.4: c.1799T>A) results in the substitution of a valine (V) to a glutamic acid (E) (p.Val600Glu), thereby inducing a conformational change into an activated form of the BRAF protein [7]. Patients with tumours that carry the BRAFV600E mutation display a less promising prognosis compared to those with a wild type BRAF, in particular, in melanoma, colorectal cancer, and thyroid cancer [19,20,21]. Since the discovery of *BRAF* mutations, efforts have been underway to understand the mechanisms by which these mutations lead to cancer progression, and to identify potential therapeutic strategies to target *BRAF* mutation positive tumours.

## 2. Involvement of BRAF in the MAPK Pathway

BRAF is part of the MAPK pathway, a signaling pathway present in all eukaryotic cells. MAPK signaling controls and regulates numerous essential cellular mechanisms including cell proliferation, differentiation, development, survival, and apoptosis [22,23,24,25]. In normal cells, extracellular signals such as cytokines, hormones, and growth factors bind to their receptor on the cells’ surface [26,27]. This binding leads to the activation of receptor tyrosine kinase (RTK) domains on the portion of the receptor on the inside of the cell. This in turn initiates activation of the kinase domain on the intracellular portion of the receptor, and a signaling cascade as sequential phosphorylation events pass from one protein to the next (i.e., RAS-RAF-MEK1/2-ERK1/2). Ultimately, a signal is passed to the nucleus, leading to transcriptional changes that promote cell proliferation. The V600E mutation in *BRAF* results in this signaling pathway being activated even in the absence of the cytokine, hormone, or growth factor stimulation, leading to unregulated cell proliferation and ultimately cancer [28].

It is worth noting that active ERK1/2 distributes a signal by phosphorylating and/or interacting with a vast array of cytosolic and nuclear substrates, each designated to initiate a specific set of functions. There are more than 150 protein substrates found in both the nucleus and the cytoplasm where activation by ERK influences transcription [27]. Therefore, there is a broad range of outcomes following activation of ERK1/2, depending on which of its broad set of targets become activated, for how long, and to what extent. A list of some of ERKs’ substrates is summarized in Table 1. Cell signaling through this pathway is not a simple on and off switch, but rather consists of multiple targets, and multiple mechanisms of feedback inhibition to modulate the pathway.

### Regulation of the MAPK Pathway

Homeostatic balance is essential for almost every physiological process in the human body. In non-transformed cells the MAPK pathway is balanced by inhibitory regulators, which provide a negative feedback signal. The MAPK pathway is in part regulated through a classical negative feedback loop, which is controlled by ERK activation of the dual specificity phosphatases (DUSPs) [58], and other molecules such as sprouty proteins (SPRYs) [59], kinase suppressor of RAS1 (KSR1) [46,60], and RAF kinase inhibitor protein (RKIP) [61,62]. DUSPs can inhibit ERK directly while SPRYs proteins inhibit the MAPK pathway at an upstream level through inhibiting RAS activation. ERK itself can also directly inactivate the MAPK pathway at several levels by directly inhibiting RTKs, RAS activation, and RAF dimerization. One of these inhibitory mechanisms is through regulation BRAF itself. Activated ERK can phosphorylate BRAF in two sites: Ser750 and Thr753, resulting in its inhibition [63]. In BRAFV600E mutations, the negative feedback mechanisms can be impaired. In some cases, the target of the inhibition is for steps upstream of the activated BRAF, and inhibition is ineffective. The mutation itself can also impair the feedback inhibition. For example, negative feedback inhibition through SPRYs is impaired because the SPRYs are unable to bind to BRAF due to disruption by mutation [64]. Constitutive activation of mutant BRAF results in hyperactive ERK that in turn can increase the expression of DUSPs and SPRYs [65]. However, this surge in expression of inhibitory regulators no longer acts as efficiently as in healthy cells.

## 3. Conferred Resistance Mechanisms in BRAFV600E Tumours

The development of drugs to target the hyperactivation of the BRAF-MAPK-ERK signaling pathway has led to substantial advances in patients’ overall survival and progression-free survival for melanoma, and the further addition of MEK inhibitors given in combination has improved response rates and survival compared to monotherapy [66]. Unfortunately, the story of BRAF inhibitors is not entirely one of success. While most melanoma cancers initially respond well to therapy, most patients will relapse with tumours that are now resistant [67,68]. For tumours other than melanoma, the combined targeted therapy is not always effective. For example, while some success with combination BRAF-MEK1/2 inhibition was observed in colorectal cancer [69], the efficacy of this combination strategy is still far less than is observed for BRAF mutant melanoma. In addition, there are some circumstances where therapy can actually result in increased tumour growth. This is a result of the inhibitors’ ability to induce a paradoxical activation of downstream signaling in BRAF-WT cells and in cell harboring RAS mutations [70,71,72,73]. Here we will discuss the main mechanisms for the resistance to therapy.

### 3.1. Resistance Through MAPK Pathway Reactivation

In the case of BRAFV600E, the reactivation of the MAPK signaling pathway accounts for the majority of acquired resistance mechanisms [74]. In a study of 100 primary and 134 follow-up samples from melanoma patients (where 87% were *BRAFV600E* positive), resistance mechanisms in the recurrent section could be identified in approximately 58% of the cases. These largely represented *BRAF* splice variants (29%) or *BRAF* gene amplification (8%) [75,76], however, secondary mutations in other genes in the RAS-RAF-MEK-ERK pathway, such as neuroblastoma RAS viral oncogene homologue (*NRAS*) [77], and MAPK/ERK kinase (*MEK*) [78,79] can lead to resistance to therapy. These mechanisms involve BRAF-independent activation of the MAPK pathway.

Secondary mutations within the *BRAF* gene have only rarely been linked to the resistance to BRAF inhibitors [80,81,82]. One exception to this is the identification of an alternative splice form of the BRAFV600E which lacks the dimerization domain and has been observed as a mechanism of resistance [83]. Resistance to BRAF inhibitors can be a result of *BRAFV600E* amplification [80]. Whole-exome sequencing of 20 melanoma patients before and after treatment with BRAF inhibitors identified that four patients with disease progression had *BRAFV600E* copy-number gain relative to baseline tumours from the same patient. Quantitative PCR confirmed an increase in BRAFV600E expression in these patients, and a cell culture model was used to demonstrate that the copy-number gain of *BRAFV600E* did indeed induce resistance to BRAF inhibitors while sensitivity was restored by its knockdown [80].

Acquired mutations in *NRAS* have been associated with acquired resistance to BRAF inhibitors. Comparing melanoma tumours collected before BRAF inhibitor therapy to resistant tumours in the same patient after therapy identified acquired *NRAS* mutations in many of these tumours, including in tumours that continue to harbor the *BRAF* mutations [84]. The Kristen rat sarcoma viral oncogene homologue (*KRAS*) mutation G12D has been identified in many tumour types, including colorectal cancers. The acquisition of this activating mutation following BRAF inhibitor exposure has been linked to the development of resistance in the BRAFV600E mutant parathyroid cancer cell line [85]. Similarly, resistance in a colorectal cell line has been linked to the appearance *KRAS* G12D and G13D mutations [86], suggesting activating mutations in this RAS pathway may contribute to intrinsic and acquired resistance. Post treatment acquisition of MEK1 and MEK2 mutations have also been associated with acquired resistance [75,86].

Besides secondary mutations to elements of the MAPK pathway, changes in gene expression level for elements of the MAPK pathway have been linked to resistance. By screening the effect of overexpressing 597 kinases, MAP3K8 (COT) kinase and C-RAF emerged as among the genes that could confer resistance to BRAF inhibitor therapy. BRAFV600E positive cancer cell lines that express higher levels of MAP3K8 tended to be less sensitive to BRAF inhibitor drugs; MAP3K8 expression increased in the tumours of patients treated with BRAF inhibitors, and was even further elevated in drug resistance relapse tumour samples [87]. Similarly, Montagut et al. found that elevated CRAF expression was observed in cells resistant to the RAF inhibitor AZ628 compared to their sensitive parental cell, and that elevated CRAF can activate the MAPK pathway independent of BRAF activity [88].

Both MAP3K8 and CRAF elevations can confer resistance either as primary or acquired resistance mechanisms. One approach that has been employed in an attempt to overcome resistance resulting from elevated expression was the use of agents that bind to and inhibit heat shock protein 90 (HSP90) [88]. HSP90 is required for the conformational stability of mutant BRAFV600E and RAF related family members [89,90,91], making blockade of HSP90 a potential strategy for overcoming resistance [92,93]. HSP90 inhibitor therapy has been included in some cancer treatment combinations [94], and has been tried in clinical phase II trials for the treatment melanoma, however, the studies either showed little effect [95] or were inconclusive [96]. Further research into this approach is required.

### 3.2. Resistance Involving Insensitivity to MAPK Regulators

Negative feedback regulators of the MAPK pathway, including DUSPs and SPRYs, have been linked to the development of acquired resistance to BRAF inhibitors. Ordinarily, a balance emerges between the activation of the RAS-RAF-MEK-ERK pathway, and negative feedback imposed by ERK-induced expression of DUSPs. Activated phosphor-ERK directly inhibits the upstream pathway, dampening the signal, and elevation in DUSPs leads to dephosphorylation of ERK, further dampening the signal cascade. Pratilas and colleagues revealed that despite elevated feedback inhibition signals, BRAFV600E is insensitive to negative feedback regulation by DUSPs [65,97]. The cell falls into a new, distorted balance with elevated ERK and elevated DUSP, but the negative feedback components are overwhelmed by persistent signaling. Similarly, SPRY2 and SPRY4 can provide negative feedback to wild type BRAF, but are unable to inhibit the BRAFV600E mutation [64]. It has been proposed that resistance to treatment may be related to further disruption in the balance between the negative feedback mechanisms and the activation [98].

### 3.3. Other Mechanisms of Resistance

The cross-talk that exists between signaling pathways activated by receptor tyrosin kinases (RTK)s, such the RAS-RAF-MEK-ERK and the PI3K-PTEN-AKT pathway, was first identified in 1994 by Chung and colleagues [99]. Overexpression of RTKs could be expected to elevate the signaling in both of these arms. Elevations in epidermal growth factor receptor (EGFR) [100], PDGFR [77,101], and IGF1-R [102] have been observed in resistance. Release of hepatocyte growth factor (HGF) from the surrounding stromal cells to activate MET, the HGF RTK on the tumour cell, has also been described as a resistance mechanism [103,104].

The integration between these two signaling pathways and the fact that both are sharing the same upstream RTKs raise the possibility of involvement of activated PI3K pathway in resistant tumour cells. Shi et al. [105] have identified BRAF inhibitor resistant melanomas with gain-of-function mutations in AKT. This AKT-mediated resistance mechanism results in P13K up-regulation. Their data suggested that, in spite of MAPK pathway inhibition through BRAF inhibitors, the BRAF mutated cells evade treatment by adapting to the use of PI3K signaling to survive. In addition to *AKT* mutations, *PTEN* mutations are found in 15.2% in metastatic melanoma leading to a similar resistance mechanism [106]. PTEN loss of function promotes AKT activation, which in turn can lead to dysregulation of the pro-apoptotic Bcl-2 like proteins. The resulting impairment of the apoptotic pathway was associated with resistance to BRAF inhibitors, vemurafenib and dabrafenib [107,108,109].

**Figure 1:**
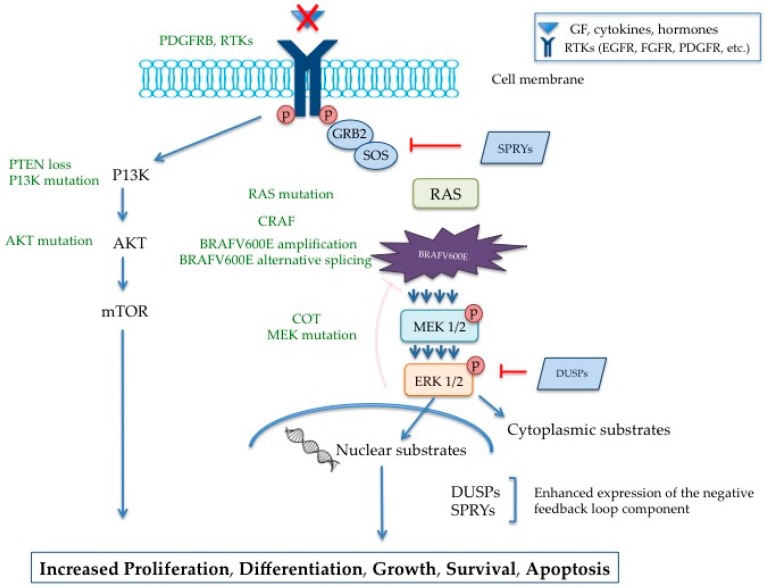
RAS/RAF/MEK aberrant signaling and mechanisms of resistance to inhibition in melanoma. Oncogenic BRAFV600E cells become independent from external growth factors (GF) (triangle symbol marked with a X) and other stimuli leading to constitutive activation of the MAPK pathway. Increased MAPK signalling (four arrows) eventually leads to enhanced gene expression including MAP kinase phosphatases (DUSPs) and sprouty proteins (SPRYs). Despite elevation of those important inhibitory regulators (T lines) of the MAPK pathway, tumour cells adapt and rely on neighbour pathways, such as the PI3K pathway, to grow and survive, Furthermore, negative inhibitory mechanisms of the MAPK pathway, including inactivation of BRAF via ERK1/2, are now lessened (faded T) due to conformational changes in the BRAF. Conferred mechanisms of resistance to BRAF inhibitors including up-regulation of PDGFRB, RAS mutations, elevation of CRAF, *BRAFV600E* amplification, alternative splicing of BRAFV600E, elevation of COT (MAP3K8), MEK mutation, PTEN loss, *PI3K* and *AKT* mutations were highlighted in green.

Figure 1 illustrates how aberrant signaling resulting from the V600E mutation in *BRAF* gene led to uncontrolled growth, and summarizes hypothesized mechanisms of resistance.

More recently, other mechanisms have been proposed. Treatment with inhibitors that inhibit MEK and ERK phosphorylation prevent the phosphorylation and stabilization of the transcriptional regulator MYC, leading to rapid degradation [110]. MYC promotes modifications to histones that influence transcription, and the loss of MYC following MEK inhibition has been found to cause epigenetic modifications to gene expression through histones and altered binding of regulatory molecules to enhancer regions [111]. While this was not specifically tested in the context of BRAFV600E resistance, this mechanism warrants consideration.

Another emerging mechanism of resistance to BRAF inhibition is through altered expression of microRNAs. MicroRNAs are small non-protein coding RNAs that bind to the transcripts of other genes and promote their degradation. Recently, the loss of microRNA miR-579-3P has been identified as a potential mechanism of both primary and acquired resistance to BRAF and MEK inhibitor drugs [112]. The mechanism by which loss of miR-579-3p leads to resistance is not fully understood, but Fattore et al. observed that this loss results in increases for both BRAF and the MDM2 pathway. MDM2 is an important negative regulator of the tumour suppressor p53, so elevation in MDM2 would reduce this protective tumour suppression pathway.

## 4. Challenges Encountered by Colorectal Cancer (CRC) Patients with BRAFV600E Mutation

The BRAFV600E mutation is found in 10% of colorectal cancer (CRC) cases [10]. Those patients progress rapidly and tend to not respond well to therapy. This subgroup of patients is distinct from other forms of CRC, and has its own molecular and genetic profile. However, the response rate to vemurafenib was only 5% in CRCs exhibiting BRAFV600E mutation compared to 60% to 80% of melanoma patients harboring the same mutation [80,106].

### Evidence of Specific Resistance Mechanisms in BRAFV600E Mutated CRC

The small subset of BRAFV600E mutant CRC displays different tumour biology and different clinical behaviors compared to *RAS* mutant CRC [113]. In a meta-analysis by Pietrantonio et al. [114], BRAF mutated CRC patients had limited benefit from any of the “available standard-of-care therapies”. These findings have raised the attention of many groups to understand why BRAF inhibitor treatment showed little or no response [67,115]. In 2012, two independent groups recognized the involvement of EGFR in CRC resistance to BRAF inhibitors. Prahallad et al. proposed that the inhibition of mutant BRAF led to a powerful feedback activation of EGFR triggering a secondary reactivation of the MAPK pathway [116]. This feedback activation of EGFR increased the activation of not only the MAPK pathway but also the parallel pathway PI3K generating growth renewal. The group studied the involvement of cell division cycle 25C (CDC25C), which is a downstream substrate of ERK that when activated can bind to and deactivate EGFR [117]. Treatment with BRAF inhibitors resulted in decreased activation of MEK1/2 and ERK1/2, consequently a failure of ERK to phosphorylate CDC25C. This failure to activate the negative feedback signal of CDC25C leads to a prolonged EGFR activation and greater activation of the P13K pathway [116]. Corcoran et al. proposed a slightly different mechanism for the prolonged EGFR activation [80]. This group postulated that negative feedback regulators such as SPRYs participated in EGFR reactivation. SPRYs comprise a key regulatory function for the MAPK pathway and transcribe in an ERK-dependent manner [118]. SPRYs negatively regulate upstream MAPK at the RTKs and RAS level. BRAF targeted therapy led to decreases in the level of SPRYs, enabling EGFR to rebound and reactivate the MAPK pathway [80]. Both groups showed that the efficacy of BRAF inhibitor is improved greatly in vitro when combined with an EGFR inhibitor and that this combined treatment leads to tumour regression in vivo. They further examined EGFR levels in clinical biopsies from patients with the BRAFV600E mutation and compared across CRC, melanoma, and PTC. The majority of BRAF mutated CRC showed high levels of active EGFR compared to other tumour types [80,116]. Moreover, single agent treatment with either inhibitor (BRAF or EGFR) produced little to poor response, indicating a combination strategy might be more appropriate for patients with BRAF mutated CRC.

Several studies that have been exploring new therapeutic approaches aimed to target resistance-conferring mutations are providing promising treatment options for patients harboring the BRAFV600E mutation. For example, Mao et al. showed that BRAF inhibitor combined with PI3K inhibitors hindered the growth of BRAF mutated CRC cell lines [119]. In addition, epigenetic factors may be playing a role in drug resistance in colorectal cancer. Hypermethylation of CpG islands is observed in colorectal tumours with the BRAFV600E mutation, and results in gene silencing of multiple target genes. Mao et al. found that the efficacy of BRAF inhibitor improves after treatment with demethylating agents [119].

Triple targeted inhibitor combinations are also being examined, combining BRAF and EGFR inhibitors with additional targets, including P13K and MEK1/2 inhibitors [120]. A more robust response rate was observed compared to monotherapy or BRAF-MEK combination therapy [121,122]. These advances illustrate the importance of understanding the underlying mechanisms of resistance in specific tumour types. New potential therapies may emerge for BRAFV600E positive CRC tumours that failed to respond to therapies designed for melanoma tumours.

## 5. Conclusions

Discovery of *BRAF* mutations in cancer allowed many scientists to link BRAFV600E with poor prognosis and overall survival in comparison to BRAF-WT and or *RAS* mutations [7,123,124,125]. The identification of this challenging subgroup of patients has shed light in the search for a broader concept of tumour progression and has helped further the investigation of therapeutic targets for cancers exhibiting the BRAFV600E mutation.

Emerging understanding of the direct and adaptive effects of BRAFV600E allow for the discovery and development of therapeutic agents that aid in reducing kinase activity of mutant BRAF and the development of strategies to overcome the resistance to such treatments. Although many individuals with recurrent melanomas or primary colon cancers fail to respond to currently available treatments, there are subpopulations that would in fact benefit from existing therapies. There has been ongoing research for robust biomarkers that can identify the activated pathways causing intrinsic resistance and acquired resistance [126]. This would potentially identify those who would benefit from treatments, and also may point the way towards preventing or reversing drug resistance.

## Figures and Tables

**Table 1:** Cellular targets of ERK.

Category	Protein	Effect of ERK Phosphorylation on Its Functions	Reference
Kinases and phosphatases	MEK1/2	Either enhances its activity or reduces it depending on the phosphorylation site	[29]
	CRAF	Inhibits its activity	[30,31]
	BRAF	Inhibits its activity	[32]
	RSK	Activation and further signal transduction	[33]
	S6K	Activation	[34,35]
	DUSPs	Negative feedback loop-indirectly via dephosphorylating ERK1/2	[36,37,38]
	SPRYs	Negative feedback loop-directly inactivating upstream	[39,40]
Signalling proteins	EGFR	Downregulation of the MAPK pathway	[41]
	Gab2 *	Reduces its activation	[42]
	SOS *	Negative feedback mechanism via preventing its association with Gab2	[43]
	IRS1 *	Impaired its downstream signalling	[44]
	TSC2	Weakens its ability to pair with TSC1, therefore Impairs its ability to inhibit mTOR signalling	[45]
Cytoskeletal proteins	Crystalline α	Anti-apoptotic protection	[46,47]
Transcription Factors	ELK *	Transcription of c-Fos	[47,48]
	c-Fos *	Acts as a sensor for ERKs’ signal duration	[49]
	c-Jun *	Transcription of c-Jun	[50]
	p53	Tumour suppressor protein, plays a role in cell cycle	[51,52]
	c-Myc *	Transcription	[41]
Apoptotic proteins	BIM *	Inhibit its pro-apoptotic function	[53]
	Caspase9	Reduce its pro-apoptotic function	[54]
	Bad *	Inhibit its pro-apoptotic function	[55]
Other proteins	RB *	Cell cycle progression	[56]
	Vif *	Activates HIV-1 replication	[57]

* Abbreviations: Gab2, GRB2 Associated Binding Protein; SOS, Ras/Rac Guanine Nucleotide Exchange Factor; IRS1, Insulin Receptor Substrate 1; ELK, ETS domain-containing protein Elk-1; c-Fos, Fos proto-oncogene; c-Jun, Jun proto-oncogene; c-Myc, v-myc avian myelocytomatosis viral oncogene homolog; BIM, Bcl-2 like proteins; Bad, Bcl-2 Associated Agonist of Cell Death; RB, retinoblastoma; Vif, Virion infectivity factor.

## Abbreviations

AKT	protein kinase B
ATP	adenosine triphosphate
BRAF	v-RAF murine sarcoma viral oncogene homolog B
CRC	colorectal cancer
EGFR	epidermal growth factor receptor
ERK	extracellular signal-regulated kinase
HSP90	heat shock protein 90
LKB1	liver kinase B1
MAPK	mitogen activated protein kinase
MEK	mitogen-activated protein kinase kinase
mTOR	mammalian target of rapamycin
PCR	polymerase chain reaction
PTEN	phosphatase and tensin homolog
RTK	receptor tyrosine kinase
TSC2	tuberous sclerosis complex 2
